# Subjective Theories of Chinese Office Workers With Irregular Physical Activity: An Interview-Based Study

**DOI:** 10.3389/fpsyg.2022.854855

**Published:** 2022-04-22

**Authors:** Borui Shang, Yanping Duan, Walter Brehm, Wei Liang

**Affiliations:** ^1^Department of Social Sciences, Hebei Sport University, Shijiazhuang, China; ^2^Department of Sport, Physical Education and Health, Faculty of Social Sciences, Hong Kong Baptist University, Kowloon, Hong Kong SAR, China; ^3^Centre for Health and Exercise Science Research, Hong Kong Baptist University, Kowloon, Hong Kong SAR, China; ^4^Institute of Sports Science, University of Bayreuth, Bayreuth, Germany

**Keywords:** physical activity, fluctuator, subjective theories, motivator, barrier, lapse

## Abstract

**Objectives:**

Individuals with irregular physical activity (PA) participation are defined as fluctuators. This study aimed to comprehend how fluctuators’ perceived barriers and motivators in their subjective theories are exhibited and cognitively represented in relation to their everyday PA practices and lapses.

**Methods:**

The design of “Research Program Subjective Theories” was used to explore and present fluctuators’ cognition concerning PA participation. Thirty fluctuators were invited to a semi-structured interview. By inductive and deductive coding, fluctuators’ verbal data were converted into word categories for extracting commonalities and comparing differences. By retaining the remaining word categories of high frequency and exploring the interrelationships among the remained word categories using statistical analyses, a superstructure (i.e., visualized representation of fluctuators’ overall cognition) including fluctuators’ main PA motivators, barriers, and behavioral outcomes was compiled.

**Results:**

Fluctuators face common motivators as barriers, such as lack of time, lack of willpower, lack of social support, and physical reasons (discomfort, injuries, or diseases). Fluctuators’ subjective theories primarily differed in motivational configurations. The physically motivated fluctuators (i.e., those predominantly motivated by physically related motivators) were more linked with low PA level, while the mixed motivated fluctuators (i.e., motivated by both physically related and emotionally or socially related motivators) were more likely associated with moderate PA level. The exemplars of the three typical fluctuators were also demonstrated to reveal their real experiences and situations in the daily life context.

**Conclusion:**

Due to the fact that fluctuation research is still in its infancy, this study represents a significant opportunity to promote knowledge growth in this area. Future studies are recommended to convert findings of the present study into interventions that benefit fluctuators in overcoming perceived barriers and enhancing motivations to eventually participate in regular PA.

## Introduction

Regular and adequate physical activity (PA) has long been linked to considerable physical and mental health benefits for individuals (Lane, 2016). The World Health Organization developed the widely accepted health-enhancing PA guidelines, which suggest that adults aged 18–64 years should accumulate at least 150 min of moderate-intensity PA per week, or 75 min of vigorous-intensity PA, or an equivalent combination, in order to reap these substantial health benefits (World Health Organization/Regional Office for Europe, 2007; World Health Organization, 2020). Despite the well-known advantages of PA, the majority of the public still finds it difficult to stick to regular PA guidelines. Some people, for instance, may follow the guidelines for a period of time, then fall into a state of inactivity (either temporary lapse or long-time relapse) for another period of time, followed by a return to PA (Kahlert, 2015).

This group of persons is referred to as “fluctuators,” and their fluctuating PA behavioral pattern is referred to as “fluctuation” (Shang et al., 2018; Duan et al., 2020).

A recent exploratory review established the groundwork for further exploration of PA fluctuation by mapping conceptual and operational concepts and presenting empirical evidence regarding fluctuation. In particular, several common characteristics of PA fluctuators were summarized in this review, such as behavioral irregularity, a significant risk of dropping out, an intention and preparedness for PA, a lack of automaticity, and inadequate self-regulation (Shang et al., 2018). Additionally, a recent study of fluctuators’ psychosocial profiles discovered that fluctuators’ mindsets might be variable. Their commitment to PA involvement differed significantly according to their demographic characteristics, such as employment position and weight status (Duan et al., 2020).

Office employees often exhibit PA fluctuations, since their daily work activities do not require substantial physical effort, resulting in sedentary behavior and a low amount of PA during work hours (Collins and O’Sullivan, 2015). Apart from their sedentary working behavioral pattern, studies reveal that office employees engage in PA on an irregular basis during their leisure time. It has also been shown that a considerable proportion of office employees are classified as PA fluctuators (Duan, 2006), and this percentage is quickly increasing (Biernat et al., 2010). Office employees with a changing PA pattern have a higher risk of developing chronic diseases, such as cardiovascular disease, musculoskeletal issues, and other chronic illnesses (Lindberg et al., 2018).

Fluctuators’ ambivalent attitudes toward PA might be the reason for their irregular participation. On the one hand, fluctuators may be motivated by the positive outcomes related to PA (e.g., health-enhancing effects and pleasure), and on the other hand, they may also be impeded by the perceived barriers (e.g., tiredness and time consumption). Such an ambivalent attitude toward PA participation may be the fundamental cause for their fluctuating behavior (Bermudez et al., 2021). Thus, a prerequisite for promoting regular PA among office-based fluctuators is a clear understanding of their motivations and barriers to PA participation. Motivations that appear particularly salient for the general adult population include improving fitness and health status, disease prevention, seeking fun and pleasure, stress reduction, and controlling weight, while common barriers are reported as lack of time, lack of social support, lack of resources, lack of interests or motivation, and discomfort or injury (General Administration of Sport of China, 2015; Herazo-Beltrán et al., 2017). However, it remains unclear as to what are the motivators and barriers for fluctuators, given that motivations and barriers differ significantly between the populations due to their subjective and perceptual nature.

One particularly promising strategy for comprehending the intricacy of fluctuators’ PA behavior and PA-related cognition is to listen to fluctuators’ “lived experiences” or “subjective theories” about their life. The term “subjective theory,” which is also used to refer to “implicit,” “naive,” “private,” or “every day” theories, means an aggregation of complex cognition expressing one’s perspective on an event or phenomenon (Grotjahn, 1991). The “Research Program Subjective Theories,” developed in Germany in the 1980s, is an exceptionally well-founded attempt to accomplish the ST, as the RPST has been widely applied in health, sports, and physical education studies (Groeben et al., 1988; Brehm and Voitländer, 2000; Weidemann, 2009; König, 2013). The philosophical rationale for RPST is critical realism’s ontology combined with an interpretive and constructive epistemology (Grotjahn, 1991; Grix, 2010). Accordingly, ontology and epistemology emphasize that human beings are not merely research subjects but are thoughtful agents with intentionality, reflectivity, potential rationality, and communicative ability (Grotjahn, 1991; Groeben and Scheele, 2001). The common design of RPST can be divided into two phases: a content extraction phase by semi-structured interview and a content construction phase by data analysis and structure-laying technique (Nolte, 2011; Flick, 2019).

The overarching aim of the present research was to increase our knowledge regarding fluctuators’ daily PA performance and the impact of perceived barriers and motivators on that performance. More precisely, we aimed to comprehend how these beliefs (perceived barriers and motivators) are exhibited and cognitively represented in relation to their everyday PA practices and lapses. In line with the research aim, the detailed research questions in this study were as follows:

1) In fluctuators’ subjective theory, what are the behavioral characteristics of their PA participation (e.g., PA type, frequency, intensity, how they organize their PA, PA interruptions/lapses)?
2) In their subjective theory, what are the motivators of and barriers to PA participation?
3) In their subjective theory, what specific reasons cause their PA interruptions or lapses?
4) What are the relationships between their PA behaviors, motivators, and barriers?
5) Are there any distinct sub-categories of fluctuators’ subjective theories? If so, how do these sub-categories differ from each other? What are the exemplars of each sub-category like?

## Materials and Methods

### Participants and Procedures

The first author of this study, a 30-year old Ph.D. candidate in exercise psychology without a personal relationship with any of the interviewees, primarily conducted the whole process of investigation. By convenience sampling strategy, all the interviewees in this research were self-reported fluctuators who volunteered to leave their personal contact in a study elsewhere (Duan et al., 2020). The self-reported fluctuators were all from office-based settings, aged 20–60 years. One of the authors contacted them and explained the study purpose, length (15–30 min), and incentive [100 RMB (15.7 USD) approximately upon completion]. Those interested were informed of the interview location and reserved time slot. Each interview was done in a secluded and quiet environment to avoid disturbance.

Thirty-three fluctuators from the previous questionnaire survey volunteered to take part in this interview. Three interviewees were omitted from the study because they claimed in the interview to have never achieved a weekly MVPA of 150 min in the preceding half-year or being not available, leaving 30 participants (14 men, 16 women) from four cities (Shijiazhuang, Xiaogan, Shenzhen, and Hangzhou) to complete the interview. Participants were aged between 22 and 56 years (*M* = 35.2, SD = 9.3). Twenty-one interviewees were married. The interviewees’ daily working time ranged from 5.5 to 10 h (*M* = 7.9, SD = 1.1). The theoretical saturation principle (Bryman, 2016) was used to determine the final sample size. Saturation was reached after little emergence of new information in the final interviews.

In line with the aforementioned research questions, the main procedures of this study involved the following steps: (1) collecting relevant information about fluctuators’ PA behavior, lapses, motivators, and barriers; (2) extracting the features based on the above-mentioned information; (3) exploring the relationships among these features; (4) classifying fluctuators into different sub-categories based on these features; and (5) demonstrating examples of each sub-category. All procedures were approved by the Institutional Review Board at one of the co-authors’ institutions. To improve the reporting transparency of this research, a checklist of Standards for Reporting Qualitative Research (SRQR, O’Brien et al., 2014) is provided in Supplementary Material 1.

### Interview Guide

A semi-structured interview guide was developed with leading questions, followed by specific probes. Four major issues were selected to correspond with the study questions: PA-related, motivator-related, barrier-related, and lapse-related (see Supplementary Material 2). Following that, sub-topics and questions were assigned to each of the major issues. Questions that addressed PA type, location, total amount (frequency, duration, and intensity), plan, implementation time, and companionship were under the PA-related subject. The lapse-related issue incorporated two sub-topics: lapse behavior (e.g., how many lapses and how long for each) and lapse causes (reason for the lapse). The sub-topics under motivator- and barrier-related topics were obtained from the Chinese Bulletin of National Physical Activity Surveillance (General Administration of Sport of China, 2015). We selected the top seven reported motivators and barriers in the surveillance for interviews to select from Supplementary Material 3. Finally, as part of the interview evaluation, a brief summary of the interview with verifying questions was provided to the interviewee to establish the validity of the interview. Upon the interviewee’s verification, each interviewee was asked to provide personal background information, such as their age, job, and marital status.

### Data Coding and Analysis

All the interviews were audio-recorded, and the important information regarding interviewees’ PA participation, motivators, and barriers was transcribed verbatim after completion. To ensure coding validity and to avoid coding subjectivity, two coders examined the raw data independently and discussed any coding discrepancies until they reached a consensus. To verify the reliability of the interview data, the coding methods were led by the coding scheme (see Supplementary Material 3), which followed multiple techniques integrating both inductive and deductive approaches recommended by Morgan (1993), Buman et al. (2010), and Flick (2019). All the inductive approaches involved familiarization with the material and grasping the general understanding through repeated reading. Indigenous typologies were then developed by carefully reading, highlighting, and rephrasing the material. If more than 10 indigenous typologies emerged, they were assigned to secondary categories by analyzing their relationships and links with each other (Morgan, 1993).

For the deductive coding, fluctuators’ responses were coded into categories according to the predefined criteria (Caspersen et al., 1985; Gollwitzer, 1999; IPAQ Research Committee, 2005). Data that cannot be deductively coded were identified and analyzed later to determine if they represent a new category or a sub-category (Hsieh and Shannon, 2005). For the scoring of motivators and barriers, the most important one is given a score of 3 points, followed by the second most important barrier is given 2 points, and the third most important barrier is given 1 point. It should be noted that some of the fluctuators’ PA-related information (PA type, location, plan, and companion) was ultimately coded at the individual level. This meant each fluctuator was regarded as an independent individual; each fluctuator was categorized into a certain category according to the information they provided. Take coding of PA type as an example, four categories were set: (1) only doing daily life PA, (2) only exercise, (3) only sports, and (4) mixed (doing at least two types of different physical activities).

The qualitative systematic aggregation method was used to develop the superstructure of fluctuators’ ST (Stössel and Scheele, 1992; Nolte, 2011). This method helps to extract commonalities among each individual’s ST and gather them in a superstructure. In this study, the process involved assessing the frequencies of coded categories from the 30 interviewed fluctuators. Only the coding categories of high frequencies and percentages (i.e., 25% interviewee reported or cited ≥5 times) were included in the superstructure. In addition, the associations between the included categories in the superstructure were explored using statistics of correlation.

The procedures for constructing superstructures with relationships between the selected categories involved the following steps: (1) re-coding the selected categories into binary variables in SPSS 24.0. For each fluctuator, each presence of the category was coded as a “1,” while each absence was coded as a “0” (e.g., assume that “lack of time” is an included category, then coding fluctuators reported it as “1,” and vice versa); (2) calculating the effect size of phi coefficients using the Chi-square test to scale the associations between binary variables; (3) summarizing all the phi coefficients into an association table. A relatively lax significance level (*p* = 0.10) was chosen to determine the inclusion of associations in the superstructure. The reason for using *p* = 0.10 instead of the conventional significance level of 0.05 was because it can help to discover more meaningful relationships between the coded categories in a small sample size but with a relatively low chance of Type I errors. Setting a significance level at *p* = 0.10 was also not rare in the previous literature (Lavrakas, 2008; Schmidt et al., 2017); (4) including relationships between coding categories lower than the predetermined significance level (*p* = 0.10) and constructing the superstructure manually. Regarding the strength of the association, *p* < 0.01 (phi coefficient of no less than 0.4), *p* < 0.05 (phi coefficients between 0.3 and 0.39), and *p* < 0.10 (phi coefficients between 0.24 and 0.29) were considered strong, moderate, and weak associations, respectively.

The sub-group differences were compared by implementing independent *t*-tests between the following five pairs: (1) men versus women; (2) younger (≤30 years) versus older (>30 years); (3) moderate PA level (≥600 MET-min/week) versus low PA level (<600 MET-min/week); (4) short period of lapse (<4 weeks) versus a long period of lapse (≥4 weeks); and (5) physically motivated versus mixed motivated. The outcome variables for these comparisons were as follows: (1) motivators (all transferred to continuous weighted score, not applicable only when comparing physically motivated and mixed motivated fluctuators); (2) barriers (all in continuous weighted score); (3) total days of lapse (not applicable when comparing fluctuators of shorter periods of lapse and longer periods of lapse); and (4) PA energy expenditure (not applicable when comparing a group of moderate PA level and a group of low PA level). Significance of *p* < 0.10 is set, and effect sizes of Cohen’s *d* are reported.

## Results

### Coding Outcomes

**FIGURE 1:**
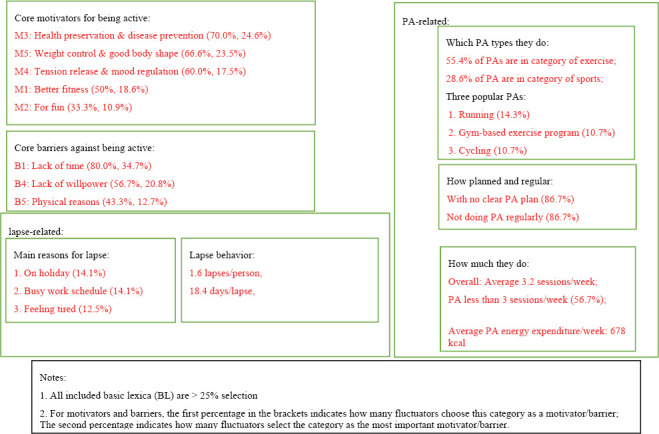
Superstructure of fluctuators’ subjective theories (*N* = 30).

Figure 1 summarizes all the key descriptive information about fluctuators’ subjective theories (cognition) in a superstructure. The main PA type of fluctuators was exercise, which contributed to 55.4% of their PA. The three most cited PAs were running, gym-based exercise programs, and cycling. On average, each fluctuator implemented 3.2 sessions of PA per week. The proportion of fluctuators who engage in PA of at least 3 sessions/week was 56.7%. According to the standard set by the IPAQ Research Committee (2005), 15 fluctuators had a low PA level, and the other 15 had a moderate level. Each fluctuator committed 1.6 lapses in the previous half-year, and on average, each lapse lasted 18.4 days. Over half (53.6%) of the fluctuators accumulated days of lapse longer than 4 weeks during the previous half-year. Fluctuators’ specific reasons for lapses were “on holiday,” “busy work,” “feeling tired,” “on a trip,” and “being lazy.”

The core motivators (>25% selection) for fluctuators’ PA were health and disease prevention (M3), followed by weight control and pursuit of good body shape (M5), tension release and mood regulation (M4), better fitness (M1), and fun (M2). The core barriers (>25% selection) preventing them from being physically active were lack of time (B1), lack of willpower (B4), physical reasons (B5), and lack of social support (B7). Regarding fluctuators’ PA plan and perceived regularity of PA behavior, 86.7% of the interviewees indicated that they had no clear plan and did not regularly perform PA.

### Exploration of Associations Among Coding Categories

**FIGURE 2:**
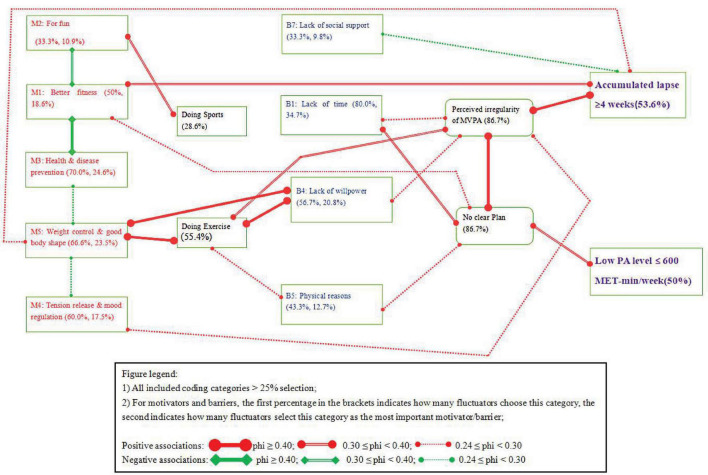
Superstructure with associations among coding categories (*N* = 30).

Table 1 presents the associations between fluctuators’ coding categories of PA behavior, motivators, barriers, and lapses, while Figure 2 visually demonstrates the significant associations. In total, 22 associations between fluctuators’ coding categories were marked. Of these, 5 associations were negatively correlated and 17 were positively correlated. Regarding the strength of the association, six associations had a phi coefficient of no less than 0.4 and were thus considered strong associations. Six associations were considered to be moderate, with phi coefficients between 0.3 and 0.39. Ten associations were regarded as weak associations, with phi coefficients between 0.24 and 0.29.

**TABLE 1:** Binary correlation table based on the coding categories in the superstructure (*N* = 30).

	M1	M2	M3	M4	M5	B1	B4	B5	B7	No-CP	PI	Exercise	Sport	Long lapse	Low PA level
M1	1														
M2	−0.36**	1													
M3	−0.45***	−0.10	1												
M4	−0.14	−0.21	−0.19	1											
M5	0.14	0.00	−0.27*	−0.29*	1										
B1	0.00	−0.04	0.08	0.10	0.00	1									
B4	0.20	−0.16	−0.07	−0.17	0.81***	0.07	1								
B5	−0.07	−0.13	0.07	0.17	0.05	−0.07	−0.05	1							
B7	−0.14	0.00	0.11	−0.14	−0.10	0.00	0.05	−0.19	1						
No-CP	0.24*	−0.10	−0.13	0.15	0.05	0.37**	0.10	0.27*	−0.13	1					
PI	0.20	−0.17	−0.02	0.28*	0.14	0.29*	0.25*	−0.05	−0.14	0.40***	1				
Exercise	0.07	−0.21	−0.23	−0.09	0.77***	0.04	0.60***	0.28*	0.00	0.10	0.39**	1			
Sport	−0.07	0.31**	0.07	0.03	−0.24	0.10	−0.05	−0.22	−0.19	0.11	0.05	/	1		
Long lapse	0.36**	0.11	−0.11	−0.10	0.28*	0.04	0.21	−0.23	−0.28*	0.24	0.44***	/	/	1	
Low PA	0.20	−0.22	0.15	0.00	−0.14	0.00	−0.07	0.20	0.00	0.34**	0.00	/	/	/	1

All scores are phi values; M1 = better fitness; M2 = fun; M3 = health preservation and disease prevention; M4 = tension release and mood regulation; M5 = weight control and good body shape; B1 = lack of time; B4 = lack of willpower; B5 = physical reasons; B7 = lack of social support; No-CP = lacking specific PA goal or situational cues; PI = perceived irregularity; LL = long lapse, lapse duration no less than 4 weeks; Low PA = <600 MET-min/week. *p < 0.10, **p < 0.05, ***p < 0.01; Blocks with “/” are not applicable for implementing correlation.

The motivators in fluctuators’ ST are mostly negatively related. The negative associations appear in four pairs of motivators:

1) Better fitness (M1) and fun (M2), phi = −0.36, *p* < 0.05;
2) Better fitness (M1) and health preservation and disease prevention (M3), phi = −0.45, *p* < 0.01;
3) Health preservation and disease prevention (M3) and weight control and good body shape (M5), phi = −0.27; *p* < 0.10;
4) Tension release and mood regulation (M4) and weight control and good body shape (M5), phi = −0.29; *p* < 0.10.

The results also show that fluctuators with the “fun” motivator were more likely to participate in sport (phi = 0.31, *p* < 0.05), while fluctuators with the motivators of weight control and good body shape were more likely to engage in exercise (phi = 0.77, *p* < 0.001).

In addition, fluctuators’ motivator of achieving better fitness (M1) was weakly associated with unclear PA plan (phi = 0.24, *p* < 0.10). The motivator of weight control and good body shape (M5) was strongly related to the lack of willpower (B4) (phi = 0.81, *p* < 0.001). Finally, the two body-related motivators, better fitness level (M1, phi = 0.36, *p* < 0.05) and weight control and good body shape (M5, phi = 0.28, *p* < 0.10), were associated with long periods (≥4 weeks) of lapse.

Figure 2 also shows that fluctuators’ barrier-related coding categories were associated with no clear plan, perceived irregularity, PA type of exercise, and longer lapses. In particular, lack of time (B1, phi = 0.37, *p* < 0.05) and physical reasons (B5, phi = 0.27; *p* < 0.10) were associated with no clear PA plan. Lack of time (B1, phi = 0.29, *p* < 0.10) and lack of willpower (B4, phi = 0.25, *p* < 0.10) were related to perceived irregularity of PA. Engaging in exercise was often related to lack of willpower (B4, phi = 0.60, *p* < 0.001) and physical reasons (B5, phi = 0.28, *p* < 0.10). Lack of social support (B7, phi = −0.28, *p* < 0.10) was negatively associated with longer duration (≥4 weeks) of lapse.

In addition, a lack of clear plan was associated with perceived irregularity (phi = 0.40, *p* < 0.01) and low PA level (phi = 0.34, *p* < 0.05). Perceived irregularity of PA was associated with PA type of exercise (phi = 0.39, *p* < 0.05) and longer periods of lapse (phi = 0.44, *p* < 0.01).

### Results From Sub-Group Comparison

The results of the sub-group comparison analysis are presented in Table 2. No significant gender difference was revealed. Regarding the age group difference, younger fluctuators (≤30 years) were less motivated by disease prevention and health preservation [*t*(28) = −2.88, *p* = 0.008, Cohen’s *d* = 1.06] than older fluctuators (>30 years). Younger fluctuators also more frequently reported “lack of time” (B1) than older fluctuators [*t*(28) = 2.28, *p* = 0.031, Cohen’s *d* = 0.85]. Finally, no significant difference was found between the fluctuators with a moderate PA level and those with a low PA level.

**TABLE 2:** Summary of significant results in *t*-tests for fluctuators’ PA motivator, barrier, and lapse.

Outcome	Group									
	*M*	SD	*n*	*M*	SD	*n*	*t*	*p*	Cohen’s *d*	*df*
		Young (≤30 years)			Old (>30 years)					
M3	0.93	0.92	14	2.00	1.10	16	−2.88	0.008	1.06	28
B1	2.50	0.94	14	1.56	1.26	16	2.28	0.031	0.85	28
		Short lapse (<28 days)			Long lapse (≥28 days)					
M7	0.62	0.96	13	0	0	15	2.49	0.020	0.91	26
B7	0.92	1.12	13	0.27	0.59	15	1.98	0.058	0.73	26
		Physically motivated			Mixed motivated					
B4	1.64	1.15	14	0.81	1.22	16	1.92	0.067	0.70	28
Total lapse days	39.46	26.72	13	24.80	13.99	15	1.86	0.075	0.69	26
PA WEE	518.7	375.4	14	817.4	563.6	16	−1.73	0.096	0.62	28

*M3 = health preservation and disease prevention; B1 = lack of time; M7 = social interaction; B7 = lack of social support; B4 = lack of willpower; PA WEE = weekly PA energy expenditure.*

Several significant differences were found between fluctuators with shorter periods of lapse (<4 weeks) and longer periods of lapse (≥4 weeks). Specifically, fluctuators with shorter lapses were more motivated by social interactions (M7) than fluctuators with longer lapses [*t*(26) = 2.49; *p* = 0.020, Cohen’s *d* = 0.91]. At the same time, fluctuators with shorter lapses were more likely to consider lack of social support (B7) as barriers than fluctuators with longer periods of lapse [*t*(26) = 1.98, *p* = 0.058, Cohen’s *d* = 0.73].

Physically motivated fluctuators were more concerned with a lack of willpower (B4) than mixed motivated fluctuators [*t*(28) = 1.92, *p* = 0.067, Cohen’s *d* = 0.70]. Physically motivated fluctuators reported longer durations of lapse [*t*(26) = 1.86, *p* = 0.075, Cohen’s *d* = 0.69] and lower MVPA energy expenditure [*t*(28) = −1.73, *p* = 0.096, Cohen’s *d* = 0.62] than the mixed motivated fluctuators.

### Categorization of Fluctuators and Exemplar Representation

To produce exemplary models from the 30 interviewed fluctuators, the first step is to classify them into distinct and representative categories. The categories were selected based on the previous results obtained in the superstructure and sub-group comparison. This study has revealed behavioral distinctions between physically motivated fluctuators and mixed motivated fluctuators. Therefore, motivator was considered as a primary factor in the classification. The 30 interviewed fluctuators were initially separated into two motivational categories (physically motivated and mixed motivated). Those in each initial category were then compared and analyzed case by case in terms of PA level, lapse duration, plan clarity, barriers, and PA regularity. By comparison and synthesis, three types of fluctuators (Types I, II, and III) were classified (see whole information in Supplementary Material 4).

Type I (physically motivated) fluctuator has the following characteristics: (1) physically motivated, (2) low PA level, (3) no clear plan for PA, (4) irregular PA participation, and (5) either lack of time or lack of willpower. Eleven fluctuators belonged to this type. Type II (mixed motivated) fluctuator has the following characteristics: (1) mixed motivated, (2) moderate PA level, (3) no clear plan for PA, and (4) irregular PA participation. Twelve fluctuators belonged to this type. Type III (impeded) fluctuator has the following characteristics: (1) mixed motivation, (2) low PA level, (3) no clear plan for PA, and (4) lack of time (caused by a critical life event, such as being a newborn baby’s mother and father). Three fluctuators belonged to this type. Four special cases could not be categorized into any of the abovementioned types and were grouped into the type “miscellany.”

As presented in Table 3, one typical case in each type (Types I, II, and III) was chosen as a representative to demonstrate the characteristics.

**TABLE 3:** Summary of exemplars.

Background information	PA information	Exemplar quotes on motivators	Exemplar quotes on barriers	Summary
27-year-old unmarried male teacher, 8 h × 5/week working (physically motivated)	Owning gym membership, swimming once per week irregularly, 2 lapses (1 and 2 months)	Motivators: better fitness, health preservation, disease prevention, and weight control. “I exercise purely to make my body better. When I was in college, my body is fit, but after graduation, my body is getting worse and my weight is going up, and my immune system also getting worse. I feel like I need a complete change, so last year I bought a gym membership card, thinking at least once a week swimming.”	Barriers: lack of willpower, physical reasons, lack of resources. “I am a bit lazy in doing exercise and not a self-disciplined guy. I am always suffering from physical discomfort after exercise such as muscle soreness and tiredness, which made me feel negative about exercise. I did not enjoy swimming particularly in hot summer when the pool is crowded and more likely to be contaminated.”	This interviewee typifies a group of fluctuators who might be not mentally ready for PA participation. These fluctuators are motivated by health-enhancing reasons (e.g., better fitness, weight control), but they do not enjoy PA. Their PA plans are easily affected by external unfavorable circumstances. As a result, they are very susceptible to long PA lapses.
31-year-old male married public servant, 9 h × 5/week working (mixed motivated)	Owning gym membership, 1 h exercise in gym (3 times/week), 1 h soccer (twice/month), 2 lapses (7 and 10 days)	Motivators: physical-related motivators (weight control, health preservation, and disease prevention), social, and emotional-related motivators (fun, tension release, and mood regulation). “I am motivated by multiple reasons, it’s really hard for me to choose only three main reasons. For me, I do gym exercise to improve my fitness and helping me release pressure, and I play soccer for meeting and chatting with my friends.”	Barriers: lack of willpower, bad weather, lack of time, physical discomfort. “The first lapse was due to a business trip and bad weather conditions. During the business trip, I thought I would compensate by doing some calisthenics, but did not implement. The other lapse was caused by catching a cold, the physical discomfort really interrupted my exercise routine.”	This individual represents fluctuators who are relatively well-prepared mentally but encounter unfavorable circumstances for PA. They are sufficiently motivated, and willing to do PA under favorable conditions. They are on the way to PA maintenance. However, they do not have a concrete plan or strong willpower to support regular implementation. Thus, when faced with various obstacles in life, they struggle to sustain regular PA.
30-year-old female public servant, married and has a 2-year-old baby (impeded)	30-min brisk walking as commuting activity irregularly (1–5 times/week); hiking irregularly (2–3 times/months), 1 lapse (6 weeks)	Motivators: better fitness, tension release, for fun and leisure. “I’m doing exercise of course for better health. I also seek for mental pleasure from doing exercise, I can experience a kind of relaxation and relief after exercise.”	Barriers: lack of time, lack of facilities. “I think lack of time is my main barrier to irregular PA participation. I need to spend tons of time to take care of my baby, especially when he got sick and other troubling issues occur. I would be more active if I could be more easily access to exercise facilities, for example, if more facilities could be set up in my community, it should be more convenient for my exercise.”	This individual illustrates how critical life evens such as giving birth and parenting can greatly affect one’s normal PA behavior, at least for a relatively short period (i.e., 6 months). For this sufficiently motivated woman, childcare demands inevitably meant that her PA engagement would be reduced.

## Discussion

To the best of our knowledge, this is the first study to explore fluctuators’ subjective theory with regard to their PA behavior, lapse, motivator, and barrier. Using the design of the research program of subjective theory, fluctuators’ verbal data from the interviews were converted into graphical representations. The fluctuators were then classified into three representative types, and each type was described with an exemplar. All the findings of this study enriched our understanding of PA fluctuation.

### Physical Activity Behavior and Lapse

This study showed that PA among the interviewed fluctuators varied greatly in both overall amount and types, which supports the hypothesized PA heterogeneity in previous research (Shang et al., 2018). Moreover, this study found that fluctuators’ PA behavior appeared to be intermittent, and more than half of the fluctuators (53.6%) accumulated more than 4 weeks of lapse. Fluctuators tended to cite more external reasons (e.g., on holiday, on a trip, overwork) for their lapses. This tendency to use external reasons (excuses) for avoiding regular PA was in agreement with the previous argument (Cohen, 2003). In addition, two frequently cited internal reasons for lapse were feeling tired and laziness. Our findings were in agreement with the findings of a previous review that work-induced fatigue is a major impediment to regular PA and exercise participation (Stults-Kolehmainen and Sinha, 2014). Research based on relapse prevention models has suggested that the internal attribution style (e.g., I am a lazy person) to the failure of a health behavior maintenance (i.e., attributing internal and uncontrollable factors to the failure) can result in prolonged lapse or relapse (Larimer et al., 1999). For future studies, the relationship between attribution style and fluctuators’ PA behavior deserves more exploration.

The study also showed that fluctuators often are planless about their PA behaviors. Half of the interviewees did not set any plan for their own PA participation, and only four formulated a detailed plan. Research has shown that planning is a critical indicator that distinguishes between PA fluctuators and maintainers (Duan et al., 2013, 2015). Helping fluctuators to formulate effective if-then plans (i.e., coping plans) involving specific responses to deal with temptation and obstacles could be an effective strategy (Gollwitzer, 1999; Sniehotta et al., 2006; Reinwand et al., 2016).

### Physical Activity Motivators and Barriers

According to the findings of this study, the common motivator in fluctuators’ ST can be differentiated into two main types: (1) physically related motivators, including health preservation and disease prevention, weight control, and better fitness, and (2) emotionally related motivators, including fun, tension release, and mood regulation. Of these two types of motivators, being physically motivated is a notable feature of fluctuators. The results might indicate that they have superficial knowledge about the benefits of regular PA engagement. In recent years, there has been an increasing amount of publicity in social and public media in China to advocate the benefits of physical exercise (Shen and Hu, 2020). In this study, the interviewed fluctuators were mostly office-based employees with high education levels. They can easily access information about the benefits of PA from various channels. We might speculate the reported physical motivators may be due to social desirability (Van de Mortel, 2008) and availability heuristic (Schwarz et al., 1991). A majority of fluctuators may not have positive affection during and after exercise. In contrast, they experienced more tiredness, discomfort, and stress.

Fluctuators face common barriers, such as lack of time, lack of willpower, lack of social support, and physical reasons (discomfort, injuries, or diseases). Lack of time is the most frequently cited barrier among fluctuators. Similar findings have been found for populations other than fluctuators during the national surveillance in China (General Administration of Sport of China, 2015), large sample surveys in the countries of the European Union (Eurobarometer, 2014), interview studies (Mathews et al., 2016; Wahlich et al., 2017), and systematic reviews (Kelly et al., 2016). Many previous research studies provided potential reasons for why lack of time is a recurring barrier among fluctuators. First, some researchers have argued that “lack of time” is a very broad notion and could serve as a pretext to hide their real barriers to being physically active (Edmunds et al., 2013; Kelly et al., 2016). Fluctuators’ lack of time for implementing PA might be simply related to low priority for PA (Cyders and Smith, 2008). Second, lack of time may reflect some fluctuators’ negative experiences in doing PA. Time perception appears shorter during pleasurable events and can appear longer during boring chores (Droit-Volet and Gil, 2009). It might be hypothesized that some fluctuators’ inability to derive joy and pleasure from PA leads them to perceive that PA participation requires large amounts of time. In retrospect, when they recall the time and experience in PA engagement, they might consider it an unpleasant and time-consuming event. The high citation frequency of “lack of willpower” among fluctuators corroborates the assumption from Fuchs (1999) that limited self-control and self-regulation regarding PA behavior are prevalent among fluctuators.

The high frequency of reported “lack of social support” coincides with the fluctuators’ dependency and the inability of PA initiation. The interview questions regarding PA organization revealed that fluctuators are not likely to be initiators of sports or exercise activities. In most cases, they are passive participants and need others to lead or supervise them to engage in PA behaviors. From the motivational perspective, fluctuators’ aforementioned passive PA participation model corresponds well to the behavioral pattern shown in people with introjected regulation in self-determination theory (Ryan and Deci, 2000; Ingledew and Markland, 2008).

### Superstructure and Sub-Group Comparison

The present study took a holistic approach to reveal many underlying interrelationships in the superstructure. Several issues are discussed in the following section. Surprisingly, no significant positive associations but four pairs of significant negative relationships were found between the five motivators. The reason might be twofold. First, these findings could be partly due to the interview design that recommended interviewees to identify 1–3 PA motivators. Therefore, once interviewees selected a motivator, the possibility of choosing the other motivators was reduced, and this might increase the chance of finding more negative associations between motivators. Second, these negative associations also reveal that in the majority of fluctuators’ ST, their health-enhancing motivators (improving fitness level, keeping weight, and preventing diseases) and motivators for being socially connected and having fun cannot co-exist simultaneously, which further agrees with the findings of previous studies targeting physically inactive individuals (Buman et al., 2010; Karssemeijer et al., 2020).

This study demonstrated the distinctions between two types (physically related versus emotionally/socially related) of motivators. Fluctuators merely motivated by long-term health-enhancing reasons were associated with unfavorable outcomes (unclear plans and prolonged lapses). Subsequently, a distinction can be made between Type I fluctuators (physically motivated) and Type II fluctuators (mixed motivated). Type II fluctuators, on average, perceived greater willpower to engage in PA behavior, committed shorter lapses, and expended more energy in PA. Previous meta-analyses showed similar findings that affective expectancies were more strongly related to PA behavior than instrumental expectancies (Rhodes et al., 2009; McEachan et al., 2016). For the unexpected results that no differences were found between fluctuators with a moderate PA level and those with a low PA level, it is possible because we asked the interviewees the following question: When you are in your physically active days, what kinds of PA you do at that time. Therefore, the amount of PA only reflects their PA in physically active periods.

Finally, special attention should be paid to a group of triadic relationships (relationships between “weight control and good body shape,” “doing exercise,” and “lack of willpower”) in the superstructure (Figure 2). This triadic relational group represents a typical mindset and related behavioral characteristics of fluctuators. First, fluctuators motivated by weight control intend to use exercise to achieve their appearance-related goals. However, changing one’s physical appearance is a very demanding process that requires physical exercise, a healthy diet, and regular rests (Donnelly et al., 2009). A substantial proportion of fluctuators do not possess effective self-control and self-regulation skills to successfully implement their PA intention and endure the unpleasant process. When exercise behaviors are implemented irregularly and fluctuators witness no obvious improvement in their physical appearance, they might start to doubt their willpower. If things continue in this vicious circle, their mindsets and irregular engagement could deteriorate into a status of fluctuating physical exercise behaviors or even utter relapse (Santos et al., 2016).

### Implications and Limitations

The findings of the present study may bear implications for PA practitioners, as well as for decision-makers in relevant organizations and policymakers in the government. Findings of the present study showed fluctuators’ common barriers, such as lack of time, lack of willpower, and physical discomfort or diseases. These findings highlight the need for a tailored intervention design from the aspect of improving time management skills (Boniwell and Zimbardo, 2004), building willpower (Oettingen et al., 2015), and preventing sport and exercise injuries (van Mechelen et al., 1993).

Moreover, the findings showed that a certain number of fluctuators are physically motivated. This implies that, at least, the previous propaganda of “exercise is medicine” and “exercise improves health” is preliminarily effective (Sallis, 2009). However, for the government, only informing people about the health benefits of PA is not enough. In the future, combining health benefits and pleasant emotional experiences (e.g., informing the benefits of improving mental well-being and adding affective elements in posters, Brown et al., 2012) may be a more effective way. It might be more comprehensive and appealing to slightly adjust the popular slogan from its original version “Exercise is medicine” to “Exercise is medicine, with delicious taste.”

Several limitations in this study are worth mentioning. First, this study focused exclusively on the perspectives of fluctuators in office settings. This neglects the perspectives of a valuable segment of fluctuators of other counterparts (e.g., technical workers, retired older adults). Second, the main characteristic of the present qualitative study is the quantification of interview-based data through the procedures of coding, synthesizing, and statistical analysis (correlation analyses and independent *t*-tests). The quantification allowed the numeric representation and manipulation of qualitative data for a more detailed description and explanation of PA fluctuation. However, the sample size cannot be considered large, and therefore the results should be interpreted with caution. Finally, it was impossible to utterly verify the authenticity of the participants’ accounts regarding their PA behavior, as well as their motivators and barriers. Future studies may employ observational or objective measures of real PA behaviors as a falsifiable criterion to improve the trustworthiness and validity.

## Conclusion

Due to the fact that fluctuation research is still in its infancy, this study represents a significant opportunity to promote knowledge growth in this area. In particular, the interview-based approach of Research Program Subjective Theories clearly illustrated how fluctuators’ perceived motivators and barriers were manifested to influence their PA behaviors and lapse. The use of this interview-based method (Research Program Subjective Theories) is suggested to get a better understanding of the PA behavior from individuals’ own perspectives and to integrate the data into theoretical models. In the future, it will be meaningful to convert the findings of the present study into interventions that would benefit fluctuators in overcoming the perceived barriers and enhancing motivations to eventually participate in regular PA.

## Data Availability Statement

The raw data supporting the conclusions of this article will be made available by the authors, without undue reservation.

## Ethics Statement

The studies involving human participants were reviewed and approved by the Institutional Review Board at Hong Kong Baptist University. The patients/participants provided their written informed consent to participate in this study. Written informed consent was obtained from the individual(s) for the publication of any potentially identifiable images or data included in this article.

## Author Contributions

YD, WB, and BS: conceptualization, methodology, and data coding and analysis. BS and YD: investigation and writing – original draft. BS, WL, YD, and WB: visualization and writing – review and editing. YD: funding acquisition. YD and WB: supervision. All authors contributed to the article and approved the submitted version.

## Conflict of Interest

The authors declare that the research was conducted in the absence of any commercial or financial relationships that could be construed as a potential conflict of interest.

## Publisher’s Note

All claims expressed in this article are solely those of the authors and do not necessarily represent those of their affiliated organizations, or those of the publisher, the editors and the reviewers. Any product that may be evaluated in this article, or claim that may be made by its manufacturer, is not guaranteed or endorsed by the publisher.
