# Supplementary material for: Subjective Theories of Chinese Office Workers With Irregular Physical Activity: An Interview-Based Study
Source: Front Psychol. 2022 Apr 22;13:854855. doi: 10.3389/fpsyg.2022.854855 (PMC9072660; doi:10.3389/fpsyg.2022.854855)
Supplement: Supplementary file 1 [file Table_1.DOCX]

# ***Supplementary Material 1***

# *STANDARDS FOR REPORTING QUALITATIVE RESEARCH CHECKLIST*

| No. | Topic | Page / line number |
| --- | --- | --- |
|  | **Title and abstract** | 1 |
| S1  S2 | Title  Abstract | 1  1 / 12-34 |
|  | **Introduction** |  |
| S3  S4 | Problem formulation  Purpose of research question | 2 / 36-77 |
|  | **Methods** | 3 / 93-104 |
| S5  S6 | Qualitative approach and research paradigm  Researcher characteristics and reflexivity | 3 / 78-92  3 / 107-109 |
| S7 | Context |  |
| S8  S9  S10  S11  S12  S13  S14  S15 | Sampling strategy  Ethical issues pertaining to human subjects  Data collection methods  Data collection instruments and technologies  Units of study  Data processing  Data analysis  Techniques to enhance trustworthiness | 3 / 109-110, 122-124  4 / 129-130  3 / 110-115  4 / 133-147  3,4 / 116-122  4,5 / 148-170  5 / 171-204  4 / 144-146, 149-153 |
|  | **Results** | 6-8 / 206-297 |
| S16  S17 | Synthesis and interpretation  Links to empirical data | Cannot be separated due to manuscript organization |
|  | **Discussion** |  |
| S18  S19 | Integration with prior work, implications, transferability and contribution(s) to the field  Limitations | 8-11 / 305-418  11 / 419-429 |
|  | **Other** |  |
| S20  S21 | Conflicts of interest  Funding | 11 / 441-442  12 / 454-455 |
