# Supplementary material for: Subjective Theories of Chinese Office Workers With Irregular Physical Activity: An Interview-Based Study
Source: Front Psychol. 2022 Apr 22;13:854855. doi: 10.3389/fpsyg.2022.854855 (PMC9072660; doi:10.3389/fpsyg.2022.854855)
Supplement: Supplementary file 2 [file Table_2.DOCX]

Supplementary Material 2

Semi-structure interview guide

**Introduction**

Hello! Please sit down. Thank you very much for sparing your time to participate this interview about your physical activity.

First let me briefly introduce myself. I am currently a PhD. student majored in physical education in Hong Kong Baptist University. This interview is a part of my dissertation. At the same time, it is also a project supported by Hong Kong Baptist University.

The purpose of this interview is to know about your daily life physical activity and your thoughts and perceptions about your PA. This interview will normally last no more than 30 minutes.

The interview is conducted anonymously and the results will remain confidential. All of your information will be only used for the sake of research. The interview content will be destroyed 6 months later after the research ends.

Besides, if you finish this interview you will be rewarded with 100 RMB. Do you have any other questions? If not, please sign this “participation agreement”. Thanks.

1. **Physical Activity -Related**

Let’s first talk about how you do physical activity in your daily life in recent half a year.

1.1 PA-participation-related

- You can think about the physical activities you normally engage in, can you tell me which physical activities you are performing? I am interested in your daily life activities as well as in your sport and exercise activities (like fitness training or competitive sports).
- Where are you normally implement them?
- Normally are you doing each of your PA (a) By yourself? (b)With friends? (c) In a group with professional instructions?
- Which of these activities are you performing in a way that you are sweating at least a bit, or you are breathing at least a bit faster? Which of these activities make you sweating a lot and even exhausted or breathless? (This question used to confirm all the activities are at least in moderate intensity)

Only the cards with at least moderate intensity are remained. And the normal walking behavior and normal housework with low intensity are removed.

- Which of these activities are you performing most often and how long is the duration of each session? What about the second most often activity and how long? On average, how often do you perform them, and how long do you do for each session?

The interviewer is writing the frequencies and duration on the cards. Then he ranks the cards with PA by frequency from most frequent PA on top to least frequent PA in the bottom.

1.2 Plan & regularity-related

- How regular is your PA participation? a) Do you have fixed dates or time for your activities or b) Are you just randomly performing PA when you have time, or performing PA following plans? If having a plan, could you tell me more about it?

The interviewer makes a note about the information regarding, plan and regularity on the cards of each type of PA.

1. **Motivators**

- Please think about why you do the physical activities above. Here I have 7 cards with reasons why people are physically active. The 7 prepared motivators are: 1) Better fitness; 2) For fun; 3) Health & Preventing disease; 4) Tension release & mood regulation; 5) Weight control & good body shape; 6) Improving skills; 7) For social interaction. Please take now 1-3 cards out of these with the motivators which are important for you and specify them.
- Do you have the motivators other than these seven? Could you tell me what are they?
- Here are all your motivators. Please rank the motivators by importance, that is, which is the most important, second and third. Could you provide brief justifications?

The interviewer writes 1-3 on the cards and is lying these cards on the left side of the PA cards with the most important on the top.

1. **Barriers**

- Please think about your own barriers for being active. Here I have seven cards with reasons why people want to be physical active but not. The 7 prepared barriers are: 1) Lack of time; 2) Lack of interest; 3) Lack of facilities; 4) Lack of willpower; 5) Physical reasons; 6) Much economic cost; 7) Lack of social support or organization. Please take now 1-3 cards with the barriers most impeding you from being regularly active.
- Do you have the barriers other than these seven? Could you tell me what are they?
- Here are all your barriers. Please rank them by importance and provide brief justifications.

The interviewer writes 1-3 on the cards and is lying these cards on the right side of the PA cards with the most important on the top.

1. **Lapses and reasons**

- Please think again about your physical activities in the **recent half a year**. Did you have a long period (at least one week or more) when you not at all implement your aforementioned physical activities (only moderate to vigorous)? How many of these interruptions did you have? How long was each interruption lasting? Could you tell me more about that?

The interviewer writes the answers on a card and lays this card under the PA cards.

- Could you tell me about the specific reasons causing these interruptions?

The interviewer makes remarks on the card according to the answers from the participant.

**Final Confirmation**

- Now it is the time to look again on the whole content you were just providing. Are you fine with the picture and whether it reflects your real thoughts? Or do you want to add, change or delete something?

If the interviewee permits, this is the final confirmation of the answers (communicative validation). If not permitted, revisions should be according made.

1. **Socio-demographic information**

I would like to end this interview with some questions about your basic information

1) How old are you? 2) What kind of job do you have? 3) How long are you working in a normal week? 4) Are you living alone or together with others? In relationship or not? Having children or not?

In the end, the researcher writes down these information as well as the interview time and location at the bottom of the card.
