# Supplementary material for: Subjective Theories of Chinese Office Workers With Irregular Physical Activity: An Interview-Based Study
Source: Front Psychol. 2022 Apr 22;13:854855. doi: 10.3389/fpsyg.2022.854855 (PMC9072660; doi:10.3389/fpsyg.2022.854855)
Supplement: Supplementary file 3 [file Table_3.DOCX]

Supplementary Material 3

Coding scheme with inductive and deductive approaches.

|  | | **Inductive coding** | **Deductive coding** |
| --- | --- | --- | --- |
| **PA-related** | | | |
| PA type | | PA types should be primarily inductively coded by counting the frequencies of indigenous typologies of PA. | If the number of primary categories by inductive coding exceed 10, PA types should be then deductively aggregated in three categories: 1) daily life PA; 2) exercise; and 3) sport (Caspersen, Powell, & Christenson, 1985). |
| PA location | | 1. Locations for PA should be firstly inductively coded by counting the frequencies of indigenous typologies.  2. If more than 10 indigenous typologies of PA locations are derived, the indigenous typologies should be further merged into secondary categories. | / |
| PA plan | | / | PA plan should be judged from two aspects (Gollwitzer, 1999):  1. Criteria specificity (aware of how much I need to do)  2. Inclusion of situational cues (e.g. when, where to do)  Two categories are generated as 1) no clear plan (involving none or only one of the previous two aspects in detail); 2) clear plan (involving both of the two aspects in detail). |
| PA regularity | |  | PA regularity directly classified into two categories (regular and not regular) directly from the interviewee’s self-report on whether they perform PA in regular time slots and locations. |
| PA amount (estimated with frequency, intensity, duration) | | / | PA frequency, intensity and duration are used to estimate the weekly energy expenditure (IPAQ Research Committee, 2005).  According to the official cutoff point, individual’s weekly energy expenditure is further classified in 3 categories:  Low PA level (< 600 MET-minutes/week);  Moderate PA level (600-3000MET-minutes/week);  High PA level (> 3000 MET-minutes/week). |
| PA companion | | / | Three categories were generated according to the interview guide: 1) by self, 2) with others (non-professional), and 3) with professional instructor |
| **Motivators & Barriers** | | | |
| Motivator | For the motivator that cannot be classified into predetermined categories, new motivator categories were generated by inductive approach aggregating the indigenous typologies. | | Originally, 7 motivator were derived from General Administration of Sport of China (2015). For the scoring, the most important motivator is scored 3 points, followed by the second most important motivator as 2 points, and third most important motivator as 1 point. |
| Barrier | For the barriers that cannot be classified into predetermined categories, new barrier categories are generated by inductive approach aggregating the indigenous typologies. | | Originally, 7 barriers were derived from General Administration of Sport of China (2015). For the scoring, the most important barrier is scored 3 points, followed by the second most important barrier as 2 points, and third most important barrier as 1 point. |
| **Lapse-related** | | | |
| Reasons of lapse | 1. Reasons for lapse should be primarily inductively coded by counting the frequencies of fluctuators’ reported reasons of lapse.  2. If more than 10 primary categories are derived, the secondary categories should be generated by further merging the primary categories. | |  |
| Lapse behavior | Based on the information of frequency and duration of lapse, total days of lapse should be calculated. Categories of long and short lapse are separated according to the median.  Equal or larger than median = group of “long lapse”;  Less than median= group of “short lapse”. | |  |
