# Supplementary material for: Subjective Theories of Chinese Office Workers With Irregular Physical Activity: An Interview-Based Study
Source: Front Psychol. 2022 Apr 22;13:854855. doi: 10.3389/fpsyg.2022.854855 (PMC9072660; doi:10.3389/fpsyg.2022.854855)
Supplement: Supplementary file 4 [file Table_4.DOCX]

Supplementary Material 4

***Categorization of fluctuators***

| Serial number | Low PA level | Long lapse duration | Clear plan | Regular PA | | B1 | B4 | B5 | B7 | Type |
| --- | --- | --- | --- | --- | --- | --- | --- | --- | --- | --- |
| Physically motivated (n= 14) | | | | | | | | | | |
| SJZ-1 | √ | √ | × | × | 0 | | 3 | 2 | 0 | I |
| SJZ-6 | √ | / | × | × | 3 | | 2 | 0 | 1 | Ⅰ |
| XG-3 | √ | × | × | × | 3 | | 0 | 1 | 0 | Ⅰ |
| XG-6 | √ | √ | × | × | 3 | | 2 | 1 | 0 | Ⅰ |
| XG-10 | √ | √ | × | × | 2 | | 1 | 0 | 0 | Ⅰ |
| HZ-1 | √ | × | × | × | 3 | | 1 | 2 | 0 | Ⅰ |
| HZ-2 | √ | √ | × | × | 2 | | 3 | 0 | 0 | Ⅰ |
| HZ-3 | √ | √ | × | × | 3 | | 2 | 0 | 0 | Ⅰ |
| HZ-5 | √ | √ | × | × | 0 | | 2 | 1 | 0 | Ⅰ |
| HZ-6 | √ | √ | × | × | 1 | | 0 | 3 | 0 | Ⅰ |
| HZ-7 | √ | × | × | × | 3 | | 1 | 0 | 2 | Ⅰ |
| SJZ-3 | × | √ | √ | √ | 0 | | 0 | 0 | 0 | / |
| SJZ-5 | × | × | √ | × | 1 | | 3 | 0 | 2 | / |
| HZ-4 | √ | × | √ | √ | 0 | | 3 | 0 | 2 | / |
| Mixed motivated (N= 16) | | | | | | | | | | |
| SJZ-2 | × | × | × | × | 3 | | 1 | 2 | 0 | Ⅱ |
| SJZ-8 | × | √ | × | × | 3 | | 1 | 2 | 0 | Ⅱ |
| SJZ-9 | × | × | × | × | 0 | | 0 | 1 | 2 | Ⅱ |
| SJZ-10 | × | √ | × | × | 3 | | 0 | 0 | 2 | Ⅱ |
| SJZ-11 | × | × | × | × | 2 | | 0 | 3 | 1 | Ⅱ |
| XG-1 | × | × | × | × | 3 | | 0 | 2 | 0 | Ⅱ |
| XG-2 | × | √ | × | × | 3 | | 2 | 0 | 1 | Ⅱ |
| XG-5 | × | × | × | × | 3 | | 0 | 0 | 0 | Ⅱ |
| XG-7 | × | × | × | × | 1 | | 3 | 1 | 0 | Ⅱ |
| XG-8 | × | √ | × | × | 2 | | 3 | 0 | 1 | Ⅱ |
| XG-9 | × | √ | × | × | 2 | | 3 | 0 | 0 | Ⅱ |
| SZ-1 | × | √ | × | × | 3 | | 0 | 0 | 0 | Ⅱ |
| SJZ-4 | √ | × | × | √ | 2 | | 0 | 1 | 3 | Ⅲ |
| SJZ-7 | √ | / | × | × | 3 | | 0 | 0 | 0 | Ⅲ |
| XG-4 | √ | √ | × | × | 3 | | 0 | 0 | 0 | Ⅲ |
| HZ-8 | × | × | √ | √ | 0 | | 0 | 0 | 0 | / |

*Note*. “√” = meeting the requirement; “×”= not meeting; “/”= not applicable or cannot be categorized; SJZ= Shijiazhuang; XG= Xiaogan; SZ= Shenzhen; HZ= Hangzhou; P=physically motivated; M= mixed motivated; Low PA level = weekly PA energy expenditure < 600 MET-minutes; Long lapse period = total lapse days ≥ 28 days; Clear plan = plan with specific goal and situational cues; B1= lack of time; B4= lack of willpower; B5= physical reasons; B7= lack of social support
